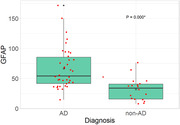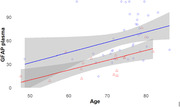# Utility of plasma GFAP as a biomarker for Alzheimer's Disease using chemiluminescent microparticle immunoassay

**DOI:** 10.1002/alz.091165

**Published:** 2025-01-09

**Authors:** Eduard Bargay Pizarro, Daniel Morell García, Ana García Martin, Susana Tarongi Sanchez, Lara Nuñez Santos, Lorena Garcia Medina, María Santés Bertó, Guillermo Amer Ferrer

**Affiliations:** ^1^ Son Espases University Hospital, Palma Spain

## Abstract

**Background:**

Glial fibrillary acidic protein (GFAP) is a putative blood biomarker for Alzheimer's disease (AD). Most studies measure plasma GFAP (pGFAP) utilizing the Single Molecule Array (Simoa) platform or other high‐cost platforms. However, we aim to validate the value of GFAP as a blood biomarker for AD using chemiluminescent microparticle immunoassay (CMIA), an ubiquitous lower‐cost platform.

**Method:**

A cohort of 56 subjects (from our memory clinic) with mild cognitive impairment (MCI) or dementia were analyzed retrospectively. Demographic and clinical data, neuroimaging, and neuropsychological test results were collected. AD cerebrospinal fluid biomarkers and pGFAP were simultaneously obtained from all patients between July 2022 and February 2023. pGFAP was measured using CMIA on the Alinity‐i‐series platform (Abbott, US), with a lower limit of quantification of 3.2pg/mL and an intra‐assay coefficient of variation <5%.

**Result:**

38 patients were classified as part of the AD continuum: 21 dementia, 18 MCI. The remaining 18 were classified as non‐AD (FTLD, LBD and non‐neurodegenerative): 5 dementia, 13 MCI. The median age in the AD group was 75.7 (IQR: 71.3‐78.2), while in non‐AD it was 71.9 (IQR: 63.3‐73.8) in non‐AD, P=0.019. There were no differences between groups in other demographic factors. We found a significant correlation between age and pGFAP increase in all patients (P=0.035). In AD group median pGFAP was 54.4pg/ml (IQR: 41.8 ‐ 85.3), compared to 34.2pg/ml (IQR: 15.8 ‐ 40.7) in non‐AD, P<0.001. These results were consistent when adjusted for confounding variables (age and other clinical and demographic variables), P<0.001. There were several trends in pGFAP levels, although not significant: higher levels in AD dementia versus AD MCI (P=0.131) and in non‐AD dementia compared to non‐AD MCI (P=0.065), also an inverse correlation between pGFAP and MMSE score (P=0.104). Using a threshold of 46.3pg/ml, GFAP alone differentiated AD from non‐AD with an area under the curve (AUC) of 0.82 (CI: 0.71‐0.94), sensitivity of 89% and specificity of 68%.

**Conclusion:**

Our findings obtained using a CMIA technique were similar to those found in the literature, in which other platforms have been used. This suggests that pGFAP measured using widely available low‐cost platform based on CMIA may have diagnostic utility for AD.